# Supplementary material for: Undergoing lignin-coated seeds to cold plasma to enhance the growth of wheat seedlings and obtain future outcome under stressed ecosystems
Source: PLoS One. 2024 Sep 24;19(9):e0308269. doi: 10.1371/journal.pone.0308269 (PMC11421780; doi:10.1371/journal.pone.0308269)
Supplement: S1 Fig — The figure shows very similar features of both spectra with slightly higher intensity at aliphatic and aromatic regions. (DOCX) [file pone.0308269.s001.docx]

*
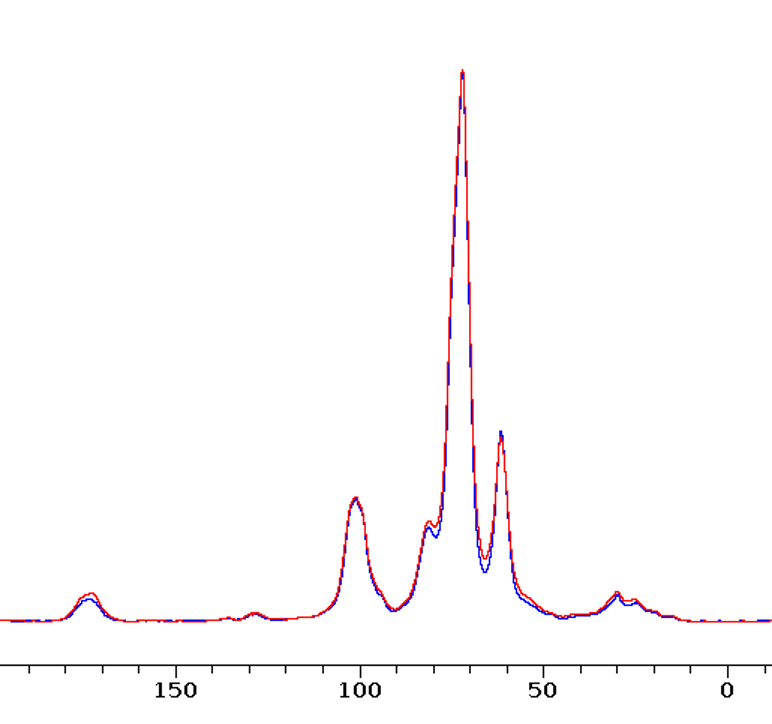
*

**S1 Fig.** Solid-state ^13^C NMR spectra of control (blue) and one-minute treated samples (red). The figure shows very similar features of both spectra with slightly higher intensity at aliphatic and aromatic regions.
