# Supplementary material for: Undergoing lignin-coated seeds to cold plasma to enhance the growth of wheat seedlings and obtain future outcome under stressed ecosystems
Source: PLoS One. 2024 Sep 24;19(9):e0308269. doi: 10.1371/journal.pone.0308269 (PMC11421780; doi:10.1371/journal.pone.0308269)
Supplement: S2 Fig — (DOCX) [file pone.0308269.s002.docx]

**S2 Fig. High resolution XPS C 1s spectra of Misr samples.**
