# Supplementary material for: Undergoing lignin-coated seeds to cold plasma to enhance the growth of wheat seedlings and obtain future outcome under stressed ecosystems
Source: PLoS One. 2024 Sep 24;19(9):e0308269. doi: 10.1371/journal.pone.0308269 (PMC11421780; doi:10.1371/journal.pone.0308269)
Supplement: Attachment — Submitted filename: PONE-D-24-08803_reviewer.pdf [file pone.0308269.s004.pdf]

PLOS ONE

Undergoing Lignin-Coated Seeds to Cold Plasma to Enhance the Growth of Wheat Seedlings and Obtain Future Outcome under Stressed Ecosystems

--Manuscript Draft--

**Manuscript Number:**	PONE-D-24-08803
**Article Type:**	Research Article
**Full Title:**	Undergoing Lignin-Coated Seeds to Cold Plasma to Enhance the Growth of Wheat Seedlings and Obtain Future Outcome under Stressed Ecosystems
**Short Title:**	Plasma as recent technology in agriculture
**Corresponding Author:**	Hani Saudy, PhD<br>Ain Shams University Faculty of Agriculture<br>Cairo, EGYPT
**Keywords:**	Agricultural wastes; harsh environments; hash carbon; lignin; plasma; wheat
**Abstract:**	Climate changes threat global food security and food production. Soil salinization is one of the major issues of changing climate, causing adverse impacts on agricultural crops. Germination and seedlings establishment are damaged under these conditions, so seeds must be safeguard before planting. Here, we use recycled organic tree waste combined with cold (low-pressure) plasma treatment as grain coating to improve the ability of wheat seed cultivars (Misr-1 and Gemmeza-11) to survive, germinate and produce healthy seedlings. The seeds were coated with biofilms of lignin and hash carbon to form a protective extracellular polymeric matrix and then exposed them to low-pressure plasma for different periods of time. The effectiveness of the coating and plasma was evaluated by characterizing the physical and surface properties of coated seeds using X-ray photoelectron spectroscopy (XPS), scanning electron microscopy (SEM), nuclear magnetic resonance (NMR) spectroscopy, and wettability testing. We also evaluated biological and physiological properties of coated seeds and plants they produced by studying germination and seedling vigor, as well as by characterizing fitness parameters of the plants derived from the seeds. The analysis revealed the optimal plasma exposure time to enhance germination and seedling growth. Taken together, our study suggests that combining the use of recycled organic tree waste and cold plasma may represent a viable strategy for improving crop seedlings performance, hence encouraging plants cultivation in stressed ecosystems.
**Order of Authors:**	Abd Elfattah Elgendy
	Hesham Elsaid
	Hani Saudy, PhD
	Nimer Wehbe
	Mohamed Ben Hassine
	Ruba Al-Nemi
	Mariusz Jaremko
	Abdul-Hamid Emwas
**Additional Information:**	
**Question**	**Response**
**Financial Disclosure**<br><br>Enter a financial disclosure statement that describes the sources of funding for the work included in this submission. Review the submission guidelines for detailed requirements. View published research	Yes

Powered by Editorial Manager® and ProduXion Manager® from Aries Systems Corporation

articles from *PLOS ONE* for specific examples.<br><br>This statement is required for submission and **will appear in the published article** if the submission is accepted. Please make sure it is accurate.<br><br>**Funded studies**<br>Enter a statement with the following details:<br>• Initials of the authors who received each award<br>• Grant numbers awarded to each author<br>• The full name of each funder<br>• URL of each funder website<br>• Did the sponsors or funders play any role in the study design, data collection and analysis, decision to publish, or preparation of the manuscript?<br><br>Did you receive funding for this work?	
Please add funding details.<br>as follow-up to "**Financial Disclosure**<br><br>Enter a financial disclosure statement that describes the sources of funding for the work included in this submission. Review the submission guidelines for detailed requirements. View published research articles from *PLOS ONE* for specific examples.<br><br>This statement is required for submission and **will appear in the published article** if the submission is accepted. Please make sure it is accurate.	The research was funded via Science & Technology Development Fund (STDF), Ministry of Scientific Research Egypt, for financial support and providing required research facilities through the approved project, ID: IG-43568.

**Funded studies**<br>Enter a statement with the following details:<br>• Initials of the authors who received each award<br>• Grant numbers awarded to each author<br>• The full name of each funder<br>• URL of each funder website<br>• Did the sponsors or funders play any role in the study design, data collection and analysis, decision to publish, or preparation of the manuscript?<br><br>Did you receive funding for this work?"	
Please select the country of your main research funder (please select carefully as in some cases this is used in fee calculation).<br>as follow-up to "**Financial Disclosure**<br><br>Enter a financial disclosure statement that describes the sources of funding for the work included in this submission. Review the submission guidelines for detailed requirements. View published research articles from *PLOS ONE* for specific examples.<br><br>This statement is required for submission and **will appear in the published article** if the submission is accepted. Please make sure it is accurate.<br><br>**Funded studies**<br>Enter a statement with the following details:<br>• Initials of the authors who received each award<br>• Grant numbers awarded to each author<br>• The full name of each funder<br>• URL of each funder website<br>• Did the sponsors or funders play any role in the study design, data collection and analysis, decision to publish, or preparation of the manuscript?<br><br>Did you receive funding for this work?"	EGYPT - EG
**Competing Interests**	The authors have declared that no competing interests exist

Powered by Editorial Manager® and ProduXion Manager® from Aries Systems Corporation

Use the instructions below to enter a competing interest statement for this submission. On behalf of all authors, disclose any competing interests that could be perceived to bias this work—acknowledging all financial support and any other relevant financial or non-financial competing interests.<br><br>This statement is required for submission and **will appear in the published article** if the submission is accepted. Please make sure it is accurate and that any funding sources listed in your Funding Information later in the submission form are also declared in your Financial Disclosure statement.<br><br>View published research articles from *PLOS ONE* for specific examples.<br><br>**NO authors have competing interests**<br><br>Enter: *The authors have declared that no competing interests exist.*<br><br>**Authors with competing interests**<br><br>Enter competing interest details beginning with this statement:<br><br>*I have read the journal's policy and the authors of this manuscript have the following competing interests: [insert competing interests here]*<br><br>* typeset	
**Ethics Statement**<br><br>Enter an ethics statement for this submission. This statement is required if the study involved:<br><br>• Human participants<br>• Human specimens or tissue<br>• Vertebrate animals or cephalopods<br>• Vertebrate embryos or tissues	NA

• Field research<br><br>Write "N/A" if the submission does not require an ethics statement.<br><br>General guidance is provided below. Consult the submission guidelines for detailed instructions. **Make sure that all information entered here is included in the Methods section of the manuscript.**	

Powered by Editorial Manager® and ProduXion Manager® from Aries Systems Corporation

**Format for specific study types**<br><br>**Human Subject Research (involving human participants and/or tissue)**<br>• Give the name of the institutional review board or ethics committee that approved the study<br>• Include the approval number and/or a statement indicating approval of this research<br>• Indicate the form of consent obtained (written/oral) or the reason that consent was not obtained (e.g. the data were analyzed anonymously)<br><br>**Animal Research (involving vertebrate animals, embryos or tissues)**<br>• Provide the name of the Institutional Animal Care and Use Committee (IACUC) or other relevant ethics board that reviewed the study protocol, and indicate whether they approved this research or granted a formal waiver of ethical approval<br>• Include an approval number if one was obtained<br>• If the study involved *non-human primates*, add *additional details* about animal welfare and steps taken to ameliorate suffering<br>• If anesthesia, euthanasia, or any kind of animal sacrifice is part of the study, include briefly which substances and/or methods were applied<br><br>**Field Research**<br><br>Include the following details if this study involves the collection of plant, animal, or other materials from a natural setting:<br>• Field permit number<br>• Name of the institution or relevant body that granted permission	
**Data Availability**<br><br>Authors are required to make all data underlying the findings described fully available, without restriction, and from the time of publication. PLOS allows rare exceptions to address legal and ethical concerns. See the PLOS Data Policy and FAQ for detailed information.	Yes - all data are fully available without restriction

A Data Availability Statement describing where the data can be found is required at submission. Your answers to this question constitute the Data Availability Statement and **will be published in the article**, if accepted.<br><br>**Important:** Stating 'data available on request from the author' is not sufficient. If your data are only available upon request, select 'No' for the first question and explain your exceptional situation in the text box.<br><br>Do the authors confirm that all data underlying the findings described in their manuscript are fully available without restriction?	
**Describe where the data may be found in full sentences. If you are copying our sample text, replace any instances of XXX with the appropriate details.**<br><br>• If the data are **held or will be held in a public repository**, include URLs, accession numbers or DOIs. If this information will only be available after acceptance, indicate this by ticking the box below. For example: *All XXX files are available from the XXX database (accession number(s) XXX, XXX.*).<br>• If the data are all contained **within the manuscript and/or Supporting Information files**, enter the following: *All relevant data are within the manuscript and its Supporting Information files.*<br>• If neither of these applies but you are able to provide **details of access elsewhere**, with or without limitations, please do so. For example:<br><br>*Data cannot be shared publicly because of [XXX]. Data are available from the XXX Institutional Data Access / Ethics Committee (contact via XXX) for researchers who meet the criteria for access to confidential data.*<br><br>*The data underlying the results presented in the study are available from (include the name of the third party*	All relevant data are within the manuscript and its Supporting Information files.

and contact information or URL).<br>• This text is appropriate if the data are owned by a third party and authors do not have permission to share the data.<br><br>* typeset	
Additional data availability information:	Tick here if the URLs/accession numbers/DOIs will be available only after acceptance of the manuscript for publication so that we can ensure their inclusion before publication.

Powered by Editorial Manager® and ProduXion Manager® from Aries Systems Corporation

Manuscript

Click here to access/download;Manuscript;Plasma manuscript.docx

Undergoing Lignin-Coated Seeds to Cold Plasma to Enhance the Growth of Wheat Seedlings and Obtain Future Outcome under Stressed Ecosystems

Abd Elfattah T. Elgendy[1], Hesham Elsaid[1], Hani S. Saudy[2*], Nimer Wehbe[3], Mohamed Ben Hassine[4], Ruba Al-Nemi[5], Mariusz Jaremko[6*], and Abdul-Hamid Emwas[7]

1 Physics Department, Faculty of Science, Ain Shams University Cairo, Egypt; e- abdelfattah.elgendy@sci.asu.edu.eg; h_m_elsaid@hotmail.com

2 Agronomy Department, Faculty of Agriculture, Ain Shams University Cairo, Egypt; e- hani_saudy@agr.asu.edu.eg; ORCID: http://orcid.org/0000–0002–6341–6156

3 Core Labs, King Abdullah University of Science and Technology (KAUST), Thuwal, Kingdom of Saudi Arabia; e-nimer.wehbe@kaust.edu.sa

4 Core Labs, King Abdullah University of Science and Technology (KAUST), Thuwal, Kingdom of Saudi Arabia; e-mohamed.benhassine@kaust.edu.sa

5 Bioscience program, Biological and Environmental Science and Engineering (BESE) Division, King Abdullah University of Science and Technology (KAUST), Thuwal 23955-6900, Kingdom of Saudi Arabia; e- ruba.nami@kaust.edu.sa

6 Smart-Health Initiative (SHI) and Red Sea Research Center (RSRC), Division of Biological and Environmental Sciences and Engineering (BESE), King Abdullah University of Science and Technology (KAUST), Thuwal, Jeddah, Saudi Arabia; e-mariusz.jaremko@kaust.edu.sa

7 Core Labs, King Abdullah University of Science and Technology (KAUST), Thuwal, 23955-6900, Kingdom of Saudi Arabia; e-abdelhamid.emwas@kaust.edu.sa

*Corresponding authors: Hani Saudy (hani_saudy@agr.asu.edu.eg;); Mariusz Jaremko (mariusz.jaremko@kaust.edu.sa)

Abstract

Climate changes threat global food security and food production. Soil salinization is one of the major issues of changing climate, causing adverse impacts on agricultural crops. Germination and seedlings establishment are damaged under these conditions, so seeds must be safeguard before planting. Here, we use recycled organic tree waste combined with cold (low-pressure) plasma treatment as grain coating to improve the ability of wheat seed cultivars (Misr-1 and Gemmeza-11) to survive, germinate and produce healthy seedlings. The seeds were coated with biofilms of lignin and hash carbon to form a protective extracellular polymeric matrix and then exposed them to low-pressure plasma for different periods of time. The effectiveness of the coating and plasma was evaluated by characterizing the physical and surface properties of coated seeds using X-ray photoelectron spectroscopy (XPS), scanning electron microscopy (SEM), nuclear magnetic resonance (NMR) spectroscopy, and wettability testing. We also evaluated biological and physiological properties of coated seeds and plants they produced by studying germination and seedling vigor, as well as by characterizing fitness parameters of the plants derived from the seeds. The analysis revealed the optimal plasma exposure time to enhance germination and seedling growth. Taken together, our study suggests that combining the use of recycled organic tree waste and cold plasma may represent a viable strategy for improving crop seedlings performance, hence encouraging plants cultivation in stressed ecosystems.

Keywords: Agricultural wastes; harsh environments; hash carbon; lignin; plasma; wheat

1. Introduction

Agricultural wastes contribute to an increase in greenhouse gases, especially CO_2, which is responsible for global warming. It has been documented that using the raw materials of plant residues as soil amendments caused increase in CO_2 and N_2O fluxes (Rizhiya et al., 2011; Toma and Hatano, 2007). There is a positive correlation between the amount of plant raw materials and gas emissions, sharing in climate changes issues (Iles, 2015). In order to reduce gaseous emissions, agricultural wastes should be converted into useful by-products. In this respect, using these by-products instead of raw wastes showed reductions in gases emissions amounted to 70% (Case et al., 2012; Felber et al., 2014; Kammann et al., 2012). Lignocellulose is the most abundant renewable raw material, but only about 4.4% of it is exploited annually (Abo et al., 2019). Lignin, a significant component in lignocellulosic materials, is an amorphous heteropolymer contains phenylpropane units linked by diversified bonds (Chandra and Madakka 2019; Geng et al. 2019). Despite there are several researches

pointed out the beneficial uses and recycling of lignocellulosic materials, knowledge about the importance of lignin as a seed coating is unavailable

On the other hand, saline soil environment is a serious challenge for agriculture in arid regions with limited access to freshwater. These regions commonly encounter harmful ecological issues such as drought, extreme heat, nutrient shortages, and soil salinization (El-Bially et al., 2022a, El-Mageed et al., 2022, El–Metwally et al., 2021, Saudy et al., 2019, Saudy et al., 2021b). Such stresses correlate with poor crop growth, development and yields (El-Bially et al., 2022b, El-Metwally and Saudy, 2021b, El–Metwally et al., 2021, Saudy et al., 2021a) and are expected to have a greater effect with global warming, risking food shortages (El-Metwally and Saudy, 2021a, Mubarak et al., 2021, Salem et al., 2021). Three main crop species, wheat (*Triticum aestivum*), rice (*Oryza sativa*), and maize (*Zea mays*) are widely grown around the world and feed more than 50% of the global population. However, present crop breeding technologies and artificial selection are insufficient to tackle the booming worldwide population and deteriorating arable farmland. To solve this issue, new strategies, such as novel smart agriculture approaches, are required to enhance the ability of agricultural plants to adapt to harsh environments.

One such emerging strategy is non-thermal plasma (NTP) or cold plasma treatment under low temperature and pressure (Elgendy, 2019, Elgendy, 2021, Elgendy et al., 2022, Elgendy et al., 2023, Khalid Buraa et al., 2019). NTP treatment is a novel class of ecologically benign methods for the secure and efficient surface decontamination and protection of crop seeds. Numerous studies have already investigated the impact of NTP on the chemistry and morphology of the seed, as well as on DNA, plant resilience to drought, and metal toxicity [Starič et al., 2020], suggesting that NTP treatment improves seed properties. Thus, plasma-treated seeds are viewed as a promising option for farming in challenging environments and soils with abnormal salinity (Alves-Junior et al., 2020, Dauwe et al., 2021, Holc et al., 2022, Pérez-Pizá et al., 2019, Shapira et al., 2019, Starič et al., 2020). However, as NTP generates a wide range of positively and negatively charged particles, and reactive oxygen and nitrogen species that may have side effects to seeds on the molecular (DNA) level, care needs to be taken to optimize plasma exposure times. On the other hand, shorter exposure times may not be sufficient to produce beneficial effects. Therefore, one of the remaining challenges in the field is the need to optimize the plasma treatment time to minimize potential unwanted effects of the NTP and decrease the costs, while maintaining the beneficial effects of seed treatment.

To address this challenge and enhance seed functionality, in the present study we made use of recycled tree waste (modified lignin and lignocellulosic material) (Söylemez et al., 2021) to be exploited in coating the wheat seed surface with a protective layer prior to NTP treatment. Lignin is a complex phenolic polymer, and the NTP treatment is expected to enhance its rigidity, the hydrophobicity of the plant cell wall, and the transport of minerals across vascular bundles in plants, thus protecting the seed from salinity. To test these hypothesizes; we focused on the analysis of two wheat varieties that are frequently used in arid regions with limited access to fresh water, *i.e.* Egyptian wheat seeds Misr-1 and Gemme-za-11. We examined how treatment with different doses of NTP affects the lignocellulosic biomaterial coating thicknesses around Misr-1 and Gemmeza-11 seeds, and how the coating thickness affects the seeds. Furthermore, after treatment, seed germination and seedling growth and physiology were measured.

2. Materials and Methods

2.1 Plant material

In every trial, we used seeds from two different wheat cultivars (Cultivar 1: Misr-1 and Cultivar 2: Gemmeza-11). As a control (D(Control)), The control seeds were not treated with chemical lignocellulosic biomass materials and plasma. The lignocellulosic biomass material (prepared as described in 2.2) treatment was done. Viscous lignin and hash carbon are mixed, and then wheat seeds are added. This material is gently mixed to create a homogeneous surface around the seeds. To protect the seeds from the wet environment, the lignin material containing hash carbon needs to be viscous and non-liquid. The seeds are left in the sun for a day to allow the surface-treatment material to dry. We start preparing the seeds for plasma exposure at different times. After the seeds were coated with biofilms of lignin and hash carbon to form a protective extracellular polymeric matrix, they were exposed to low-pressure plasma. The low-pressure plasma treatments were done over one of four time periods: one minute (D(00)), two minutes (D(22)), three minutes (D(33)), or four minutes (D(44)), as described in 2.3.

2.2 Chemical lignocellulosic biomass materials

Lignocellulosic biomass in crushed form (roughly ~4 mm in size) was purchased from a mill and then sieved to produce a powder with particle sizes of 250-500 μm. Flavone ligninocellulose (FLC) was prepared by a one-pot reaction method (Davin and Lewis, 2005, Ge and Li, 2018, Liu et al., 2019). Co-monomers including acrylamide and vinyl from treated–grafted polymer were purchased from Sigma-Aldrich as a compatibilizer (Boerjan et al., 2003). All other chemicals were obtained from commercial sources and were of reagent grade. For the nano-fibrillation of FLC powder, 10 g of untreated LC powder was soaked in distilled water for 48 h. The suspension of LC powder (1 wt%) was then agitated using a high-speed blender (Vitamix TNC5200, Vita-Mix Corporation, Cleveland, OH, USA) equipped with a 2 L SUS container (X-TREME CAC90B, 143 WARING, East Windsor, NJ, USA) at a stirring speed of 37,000 rpm. After stirring for 45 min, nanofibrillated fiber was obtained by centrifugation at 8,000 × g for 20 min at 25°C. The recovered wet fiber was infiltrated with t-butyl alcohol and freeze-dried (Boerjan et al., 2003, Davin and Lewis, 2005, Ma et al., 2020).

For the pretreatment of FLC powder, 10 g of ligninocellulose (LC) was mixed with 10 g of 3.4-dihydroxyacetophenone and 10 mL dimethyl sulfoxide (DMSO as cosolvent) and heated in a dry oven at 110°C for 16 h. The reaction mixture was then suspended in 100 mL of deionized water and centrifuged at 8,000 ×*g* for 20 min at 25°C. This washing step was conducted five times to remove the 3.4-dihydroxyacetophenone/DMSO solution. The product was dried in an oven at 90°C for 24 h and grounded into a powder using a mill.

For alkylation, 5 g of bagasse sample was mixed with 10 g of chloroacetic acid in a 100-mL pressure-proof autoclave reactor (microwave synthesis and extraction, MW-US-UV syn, Co. Ltd., Nagoya, Japan). The reactor was heated in a rotary oven at 140°C. After heating for 3 h, the modified FLC was washed three times with acetone and then twice with distilled water. To remove unreacted chloroacetic acid, FLC acetic acid was centrifuged at 13,000 × *g* for 20 min at 25°C followed by removal of the supernatant. To prepare the composites, a mixture of 18.8 g of thiosemicarbazide and 1.2 g of FLC acetic acid in 500 mL of xylene was stirred at 130°C for 1 h to obtain a homogeneous solution. The suspension was cast onto a tray covered with

polyamide film. After the evaporation of xylene at room temperature in a well-ventilated hood, the resultant dry mixture (thiadiazole biofilm of FLC sample = 47:3:50) was cut into pieces and compounded using a co-rotating twin-screw micro extruder with a recirculating channel (MC5, Xplore Instruments, Sittard, Netherlands) for 3 min at 180°C and 60 rpm. The extruded strands were cooled in air and pelletized. The pellets were injection-molded into dumbbell-shaped specimens (JIS K7161) (Japanese Standards Association, 2014). The injection and mold temperatures were 190°C and 100°C, respectively (Baraka et al., 2023, Gao and Fatehi, 2019, Ge and Li, 2018, Liu et al., 2019, Ma et al., 2020, Nassar et al., 2023).

2.3 Plasma treatment

An alternating current (AC) plasma system was used to coat the seeds. The device generates reactive ion species in gases such as argon, helium, oxygen, and nitrogen at low temperature under a low vacuum (25-90 Pa). Air gas can produce different reactive species like nitrogen, oxygen, carbon and OH groups to enhance the surface of the coated seeds and protect it (Elgendy, 2019, Khalid Buraa et al., 2019).These species react with the seed's surface to generate different chemical entities that can act as a fertilizer and protectant, including making the seeds more resistant to salinity. In addition, using air gas under low vacuum is more chemically effective and considerably lowers the cost when considering larger scale production. The physical parameters of the AC plasma discharge were pressure $(p)\ = 25 - 90\ \text{Pascal},\ T_G = 300\,K,$ applied voltage $(V(t)) = 400\ \text{Volt}$, and ion current flux $(e\psi_i) = \cdots 0.5..\ \text{mAm}^{-2},\ T_e = 3\ \text{e}V$. Power (P) was set to 41-50 W.

The ion flux and ion energy of the reactive species, such as nitrogen, oxygen, and helium, can be detected. The ion flux and energy (Eq. 1 and 2) were calculated using the following equations.

Ion flux: $\Gamma_i = 0.61 n_0 \sqrt{T_e/m_i}$ (1)

Ion energy: $\varepsilon_i = \frac{eT_e}{2} \ln(\text{M}/(2.3\ m_e))$ (2)

where $\boldsymbol{n_e}$ is plasma density, $\boldsymbol{T_e}$ is thermal energy, and M is the ion mass of the reactive species. The ion energy of reactive air species in our study such as nitrogen, and oxygen work at lower energy (2.1 - 5 eV) which may be more effective for soft biological treatment and not harmful for our seed treatments.

2.4 Wettability measurements

For wettability measurements, 1 □L water droplets were placed on the wheat grain surfaces, and brightfield images of all treated and untreated grains in the same frame were taken using a standard stereomicroscope. The diffuse area of the droplets on the surface indicates wettability.

$\gamma_S = \gamma_L . \cos\theta + \gamma_{SL}.$ (3)

Where, γ_S: is the solid surface tension, γ_L: is the liquid surface tension, γ_{SL} : A solid -land boundary surface tension:

2.5 Scanning electron microscopy (SEM)

The morphology of the seeds was studied using a Quattro ThermoFisher SEM equipped with a field emission gun (FEG). SEM images were recorded at an accelerating voltage of 5 kV using a secondary electron detector after sputter coating the seeds.

2.6 X-ray photoelectron spectroscopy (XPS)

To determine the elemental composition and the chemical state of the seeds, an XPS system (Kratos Axis Supra equipped with a monochromatic Al Kα X-ray source (hν = 1486.6 eV) operating at a power of 75 W and under UHV conditions in the range of ∼10−9 mbar was employed. All spectra were recorded in hybrid mode using electrostatic and magnetic lenses and an aperture slot of 300 µm × 700 µm. The wide and high-resolution spectra were acquired at fixed analyzer pass energies of 80 eV and 20 eV, respectively. The samples were mounted in floating mode to avoid differential charging.

2.7 Nuclear Magnetic Resonance (NMR) spectroscopy

All NMR spectra were recorded using a Bruker 400 MHz AVANACIII NMR spectrometer equipped with 4 mm Bruker MAS probe (BrukerBioSpin, Rheinstetten, Germany). The seeds were ground to fine powder using an electrical grinder, then the grain powder was packed in to 4mm zirconium oxide MAS rotor. To create comparable data all experiments were recorded under the same conditions using the same instrumental parameters described previously (Alkordi et al., 2015, Chisca et al., 2014). The data acquisition and analyses were performed using Topspin 3.5pl7 software (Bruker BioSpin, Rheinstetten, Germany).

2.8 Germination and seedling vigor test

Two tested cultivars (Misr-1 and Gemmeza-11) were placed on 15 cm diameter petri lined with filter paper (#1 Whatman International, Maidstone, UK) as the media. The filter paper was initially moistened with 7 mL of distilled water, and an additional 5 mL of water was added on the fifth and ninth days of the experiment. Four plasma exposure times were applied plus the control (D(control), D(00), D(22), D(33), and D(44)). For each cultivar, the plasma treatments were arranged in a completely randomized design with four replicates in ambient laboratory conditions (20 ±1 ºC). Full germination was obtained for all tested treatments (100%). After15 days, the seedlings were isolated to measure the radical length, plumule length and seedling length. Seedling dry weight was recorded after oven drying at 105 ºC for 24 h.

2.9 Greenhouse trial and physiological studies

At the Faculty of Agriculture, Ain Shams University, the plasma-treated wheat seeds were planted in pots using the randomized complete block design in four replicates. On 2 December 2021, five seeds of wheat cultivars Misr-1 and Gemmeza-11 were placed in plastic pots of 30-cm diameter and filled with 6 kg soil and subjected to the above plasma doses. At 60 days of age, plant leaf samples were taken to estimate chlorophyll a (ch-a), chlorophyll (ch-b), total chlorophyll, carotenoids, malondialdehyde (MDA) and hydrogen peroxide (H_2O_2) levels following previous reports (Arnon, 1949; Madhava Rao and Sresty, 2000; Rizk et al., 2019; Velikova et al., 2000).

2.10 Statistical analysis

Data were subjected to an analysis of variance (ANOVA) test according to Casella (2008), using Costat software, version 6.303 (CoHort Software, Monterey, CA). Based on the randomized complete block design in four replicates, plasma treatment and wheat cultivar were considered as fixed effects, and replications (blocks) were considered as random effects. Mean separation was performed only when the F–test showed significant ($P \leq 0.05$) differences among the treatments based on Duncan's multiple range test.

3. Results

3.1 Nonthermal Plasma Composition

To test the properties of the NTP used during the coating process, we performed spectroscopic analysis of the air during coating at different applied voltages. This analysis revealed that, as expected, higher voltage levels generated higher levels of ionization, resulting in higher emission intensity (Figure 1, left panel). Furthermore, we also observed, as expected, that higher voltage led to a higher concentration of reactive species such as nitrogen, oxygen, and iron in our plasma (Figure 1, right panel). Based on these results we conducted all our NTP treatment experiments using 400 V, as these conditions corresponded to suitable reactive species that could interact safely with seeds

3.2 Effect of plasma dose on wheat seedling growth rate, germination and vigor

Next, we tested the effect of the NTP dose on the growth status of wheat seedlings. We evaluated four different NTP treatment times (1, 2, 3 and 4 minute exposures), and compared those results to the ones obtained under "no treatment" conditions. As described in Methods and Materials, seeds of each cultivar (Misr-1 and Gemmza-11) were grown under laboratory conditions for 15 days. The seedling growth was measured at regular intervals, and calculated at day 15 (Table 1). We also calculated a correlation coefficient to evaluate the linear relationship between NTP does time and the seedling growth rate (Table 1). We observed that 2 minute NTP treatment produced optimal results in both cultivars. We also noted that 1 minute treatment was not sufficient to reach the maximum beneficial effect (seen at 2 minutes); however, even the 1 minute treatment resulted in some improvement over "no treatment" conditions. Additionally, both 3 and 4 minute treatments led to some deterioration of growth rates, when compared to the 2 minute dose. We also observed some possible differences between the two cultivars, given that Misr-11 exhibited differences between 3 and 4 minute treatments, whereas Gemmeza-11 did not. These results suggest that conclusions based on one cultivar may not translate to others, and that each cultivar needs to be individually evaluated to determine the most optimal treatment conditions.

To further examine the effects of different NTP treatment doses on wheat seeds and their ability to germinate and produce a healthy seedling, we also examined seed germination and seedling vigor. Therefore, five seeds of each wheat cultivar treated with different plasma conditions were planted in petri dishes. After one-month, radical length, plumule length, seedling length and seedling dry weight were measured. Figure 2 show such parameters for Misr-1 and Gemmeza-11 cultivars, respectively. For Misr-1, just the treatment with lignocellulosic biomass (D(00)) was sufficient to result in increases in radical length, plumule length, seedling length and seedling dry weight of 7.9%, 18.1%, 12.0% and 19.7%, respectively (Figure 2A shows just the effects on seedling length and dry weight; results regarding radical length and plumule length are shown in Supplemental Information, as is the Table 1 with exact values from which we derived the % values mentioned in

the text). In these experiments with Misr-1, the best increases under NTP dosing conditions were obtained with D(33) (1.2%, 25.0%, 10.9% and 28.1%). For Gemmeza-11 (Figure 2B), we observed more dramatic effects on seedling length, as D(00) increased the values by 51.4%, 3.9%, 29.3% and 12.0%, respectively, and D(22) increased them to 46.2%, 9.0%, 29.0% and 19.7%. For both cultivars, D(44) was detrimental for their growth, and, additionally, Gemmeza-11 appeared to be especially sensitive to optimal treatment. Taken together, these studies showed that combined treatment of wheat seeds with recycled tree waste (lignocellulosic biomass) and low-pressure plasma (NTP) has the potential to improve seedling growth rates, germination and vigor.

3.3 Wettability

To identify potential reasons for the improvements which observed in our seedling studies, the seed surface wettability, a very important parameter for germination, was assessed. Thus, improving and increasing the seed surface wettability is a desirable step in seed treatment approaches. Thus, we conducted seed wettability experiments to examine whether our seed treatment strategy had in impact on this parameter. The contact areas of 1 □L size water droplets with seeds treated at different NTP exposure times are shown for Misr-1 in Figure 3A and for Gemmeza-11 in Figure 3B (see also Table 1). Misr-1 seeds treated with a 2 minute NTP dose (D(22)) had reduced wettability compared to D(control) and D(00). In contrast, D(33) or D(44) treated samples had increased levels of surface wettability. On the other hand, Gemmeza-11 seeds had fluctuating wettability levels. The more fluctuating results of the Gemmeza-11 show that its higher sensitivity to plasma treatment when compared to Misr-1, which is aligned with what we observed above in our seedling experiments.

To quantify the seed wettability, we calculated the values of water droplet contact areas on the surface of controlled and plasma treated seeds Misr-1 and Gemmeza-11 (Table 1). In agreement with our microscopy based observations, we noted that the water/seed contact area is significantly decreased in Misr-1 under D(22) dose conditions, and that effects in Gemmeza-11 are much more variable across the NTP treatment conditions. From these observations, we concluded that the exposure time affects wettability and nutrient accumulation. Notably, D(00), D(22), and D(33) had the strongest effect on both Misr-1 and Gemmeza-11 cultivars. However, correlating wettability with other seedling traits, including radical length, plumule length, seedling length, and seedling dry weight is less clear.

3.4 Solid State Nuclear Magnetic Resonance (NMR) characterization of treated seeds.

We wanted to examine whether our combined treatment with recycled tree waste (lignocellulosic biomass) and low-pressure plasma (NTP) led to specific structural and chemical changes on the seed. For overall seed chemical composition evaluation, we chose to apply solid-state Nuclear Magnetic Resonance (NMR) spectroscopy. NMR spectroscopy is a powerful analytical tool that has been extensively used to study chemical composition and molecular identity (Emwas et al., 2020, Kwan and Huang, 2008). The main advantages of NMR spectroscopy are that it is a nondestructive and highly reproducible method where samples can be studied in both solid and liquid states (Chandra et al., 2021a, Chandra et al., 2021b). In the current study we employed solid state NMR spectroscopy to compare the selected treated seeds with the control ones. Given that solid state NMR has not previously been used for wheat seed analysis, we had to develop a sample preparation procedure that would retain seed integrity and allow for accurate acquisition of the NMR data (see sample preparation description in Materials and Methods section). As seen in supplementary materials Figure 1, the seed samples

yielded well-disperse solid-state NMR spectra, clearly showing the distribution of the signals corresponding to chemical nature of carbon (aliphatic region between 0 and 100 ppm; aromatic region above 150 ppm) (Li et al., 2016, Khan et al., 2014). We noticed that the treated sample (D(11) in red; supplementary materials Figure 1) displayed a slight increase in NMR peak intensity in aliphatic region where signals from alkyl groups can be found (0-50 ppm) and in the aromatic region (around 175 ppm) when compared to the control (in blue; supplementary materials Figure 1). These small differences in the peak's intensity might be associated with the coating layer of lignin on the surface of the treated wheat seeds, which may improve seed resistance to pathogens or drought, as described before, and is in agreement with improvements we described above.

3.5 SEM analysis of Misr-1 and Gemmeza-11 cultivar seeds

To investigate whether the treatment changed the structure of the seed's coat in a manner that may explain improvements in seedling growth rates, germination and vigor, we used scanning electron microscopy (SEM). This technique allowed us to visualize the finer structural detail of the wheat seed surface, and compare the features of the two cultivars under different treatment conditions. Figure 4 shows SEM images of the Misr-1 samples, including the untreated seed surface at different scales. For D(00), a very thin coating is observed at the top of the seed with 1-10 µm particles (arrows in Figure 4b-b''). For D(22), a coating of about 30-µm thickness covers almost the whole seed (arrows in Figure 4c-c''). D(33) resulted in a thin coating covering a few hundred micrometers of the seed surface (Figure 4d-d''). For D(44), the coating is barely seen, and only some small particles of a few micrometers in size are observed at the top surface (Figure 4e-e''). Therefore, we conclude that for Misr-1, D(22) conditions produced the most extensive coating, which may explain the superior seedling growth rate that we noted above. Figure 5 shows SEM images of Gemmeza-11 samples. For D(00) and D(44), a significant coating of about 50 µm was identified at the top surface of the seeds (arrows in b-b'' and e-e''). For D(22) and D(33), the coating was very thin, and only a few particles of about 1 µm in size were present (Figure. 5c-c'' and d-d''). Therefore, for Gemmeza-11, the link between coating and the results of the seedling growth tests do not seem to be clear at this point. Moreover, these results further highlight the cultivar-dependent response to the coating process.

3.6 XPS analysis of Misr-1 and Gemmeza-11 cultivar seeds

Lastly, to gain deeper insights into how the treatment changed the chemical composition of the seeds' surface, we employed X-ray photoelectron spectroscopy (XPS). XPS is a technique that allows elemental analysis of surfaces with high sensitivity. XPS spetra of Gemmeza-11 samples indicated that all seeds have a very similar composition independent of NTP exposure time (Figure 6). However, compared to the control, samples exposed to NTP showed a slight decrease in carbon content and an increase in oxygen content. Also, there was a significant decrease in N content for D00 with respect to other samples. In terms of the elemental content, C, N, and O elements account for about 95-98% of the composition of each sample irrespective of NTP exposure time, and for 99% of the untreated sample. NTP led to the inclusion of several elements on the wheat surface, but the overall concentration of these elements remained relatively low. The chemical structure of Gemmeza-11 samples did not undergo significant changes following NTP exposure according to the C 1s high-resolution spectra (See supplementary materials Figure 2 and 3), whose peak shape was very similar for all samples. The

main carbon bonds assigned to C-N, C-O (detected at around 286 eV), C=O and O-C=O (detected at around 288 eV) exhibited similar shapes and intensities independent of the NTP exposure.

In comparison, Misr-1 showed some different trends (Figure 7). Again, the main composition of NTP-exposed samples was more than 97% C, N and O, and 100% for the control seed indicating potential variability in sed surface composition between cultivars. However, there was some variations in the C/O ratio after exposure to NTP. Misr-1D(00) had the highest C/O ratio (and thus the lowest oxygen content), whereas Misr-1D(22) had the lowest. The spectra also confirmed the insertion of several elements at low concentration to the wheat surface following NTP exposure. In contrast to Gemmeza-11, where the chemical structure was very similar for all samples irrespective of the exposure, the Misr-1 samples showed clear dose-dependency (**Figure 7**). The main chemical bonds assigned to C-N, C-O (detected at around 286 eV), C=O and O-C=O (detected at around 288 eV) were less pronounced for samples D(00) and D(44) compared to other doses. This observation is consistent with the lower O and N content of both samples, as shown by the atomic percentages shown in the XPS spectra (Figure 7).

3.7 Greenhouse trial and physiological studies

Finally, we wanted to examine how different treatments affect the development and physiology of the resulting plants. Based on the above observations, we selected to test seeds from both cultivars (Misr-1 and Gemmeza-11) and expose them to three plasma doses (D00, D22 and D33). We planted the seeds as described in Materials and Methods and grew them under greenhouse conditions as specified. We harvested the leaves after 60 days, and analyzed their chlorophyll a (ch-a), chlorophyll (ch-b), total chlorophyll, carotenoids, malondialdehyde (MDA) and hydrogen peroxide (H_2O_2) levels. As shown in Table 2, D(00)-treated Misr-1 cultivar seeds showed the most ch-b, D(22) showed the most ch-a, and D(33) showed the most H_2O_2. For Gemmeza-11 cultivar, D(22) showed the highest content of carotenoids, MDA and H_2O_2, and D(33) resulted in the highest values for ch-a, ch-b and total chlorophyll. NPT treatment showed positive effects on photosynthetic pigments (ch-a, chlorophyll, total chlorophyll, and carotenoids), as well as enzymatic-protective antioxidants (malondialdehyde and hydrogen peroxide). Future open-field trials should be carried out under various abiotic stresses, such as salinity, to assess the effects of plasma on seed robustness.

4. Discussion

Plasma exposure can change seed-surface properties and stimulate seed germination and seedling growth, induce changes in metabolic plant pathways, modulate enzymatic activities and phytohormones, induce stress resistance, and ultimately influence crop productivity (Han et al., 2016, Holc et al., 2021, Jeevan Kumar et al., 2015, Mildaziene et al., 2021, Scholtz et al., 2021, Šerá et al., 2021, ten Bosch et al., 2017). Plasma treatment affects the wettability of the coated seed. Investigations of plasma-induced changes in plant physiology and biochemistry are likely to reveal new facts of fundamental and applied interest (Mildaziene and Sera, 2022). Our findings indicate remarkable responses of wheat seeds and seedlings to plasma. Since the glow discharge of cold plasma has ions that cause strong surface etching, plasma treatment of wheat seeds (Misr-1 and Gemmeza-11) resulted in chemical re-structuring of the coated surface. In addition, glow plasma contains a higher concentration of relatively aggressive reactive chemical species of higher energy as well as vacuum ultraviolet

radiation, which may explain why variations in the nutrient accumulation on the seed coat surface we observed depended on the plasma treatment.

The results indicate also that the response of seeds and seedlings to plasma process cannot be generalized. This is because every seed has its own structure, and so exposure to the same conditions will have different effects. Additionally, each wheat cultivar has distinct coping mechanisms and interactions with highly reactive oxygen plasma species. Therefore, the different observed responses to plasma could be attributed to the diversified chemical structures of the seed surface and seed size, in addition to the genetic makeup (Zahoranová et al., 2016). The seedling growth rate measurements showed that Gemmeza-11 cultivar is more sensitive than Misr-1 to plasma treatment. In this context, the Gemmeza-11 seedling length and weight were lower after plasma treatment for 3 min based on the reduction in MDA and H_2O_2 levels. Misr-1 showed better responsiveness and adaptability to cold plasma treatment. Our findings revealed that seed properties can be optimized by optimizing plasma dose to ensure that the coating treatment matches the needs of a specific cultivar. Further studies are needed to examine whether different attributes (*i.e.* resistance to bacteria or resistance to drought or salinity) require treatment with different plasma doses.

In addition to investigating the effects of the NTP treatment dose on wheat seeds, we also co-treated the seeds with a low molecular-mass biopolymer containing lignin-cellulose. This biopolymer had a major impact on the wetting-drying cycle of the wheat seeds tested here. In previous studies, after 10 wetting–drying cycles, water stability increased 1.5-20 times depending on the soil. The addition of a low molecular-mass biopolymer containing lignin-cellulose exposed under D(22) after a single wetting-drying cycle increased the water stability more than 200 times. Wettability is not the only factor that influences seed germination. Others include pH, salt concentration, osmotic potential, C, O and N content, bulk density, porosity, and particle size distribution, and these should be considered in lignin-cellulose biofilm design.

Finally, plasma treatment is known to improve the surface hydrophilicity and water uptake of seeds. These effects are mediated by the functionalization and etching of the seed surface (Attia et al., 2019, El-Hashash et al., 2018, El-Hashash and Rizk, 2017, Rizk et al., 2019, Rizk et al., 2018). We observed these effects on the seed surface structure using SEM analysis, which showed that a significant coating of lignin is around 30 µm. For Misr-1, NPT at D(00) and D(22) resulted in resistance to soil salinity. A thinner thickness required a longer plasma dose (D(33)), but too long a dose (D(44) and D(55)) resulted in less coating and less resistance to salinity. For Gemmeza-11, a coating of about 50 µm plasma was effective at D(00) and D(22).

5. Conclusions

The current work presents the first report of combining the use of organic materials extracted from tree waste residues with plasma treatment for seed coating. This seed coating approach offers many advantages for seeds. The coating provides a protective layer that helps adaption in harsh conditions such as high salinity. The organic layer also helps to absorb water and preserve it around the seeds, which helps in germination. Also, this layer forms nutrients for the plant. It acts as a fertilizer for the plant and a food for the bacteria that secrete nutrients for the plants. Thus, the treating seed with both lignocellulose and cold plasma may contribute to the production of seeds robust against undesirable environmental conditions, and open opportunities for the mass cultivation of strategic crops like wheat in stressed environments such as saline soils. Further investigations should be adopted to apply the promising findings of plasma plus lignin coated seeds under open field experimentation.

Declarations

Ethics approval and consent to participate

Not applicable

Consent for publication

Not applicable

Availability of data and material

The datasets generated during the current study are available from the corresponding author on reasonable request.

Competing interests

The authors declare no conflict of interest.

Funding

The research was funded via Science & Technology Development Fund (STDF), Ministry of Scientific Research Egypt, for financial support and providing required research facilities through the approved project, ID: IG-43568.

Authors' contributions

Conceptualization, Elgendy A.T, Elsaid H., Saudy H.S., Ben Hassine M., Al-Nemi R., Emwas A..; methodology, Elgendy A.T, Elsaid H., Saudy H.S., Wehbe N., Ben Hassine M., Al-Nemi R., Jaremko M., Emwas A.; software, Wehbe N., Ben Hassine M., Al-Nemi R., Jaremko M., Emwas A.; validation, Elgendy A.T, Saudy H.S., Wehbe N., Ben Hassine M., Al-Nemi R., Jaremko M.; formal analysis, Elsaid H., Saudy H.S., Jaremko M., Emwas A.; investigation, Elgendy A.T, Elsaid H., Saudy H.S., Emwas A. ; resources, Elgendy A.T, Saudy H.S., Wehbe N., Jaremko M., Emwas A. ; data curation, Elgendy A.T, Elsaid H., Saudy H.S., Wehbe N., Ben Hassine M., Al-Nemi R., Emwas A.; writing—review and editing, Elgendy A.T, Elsaid H., Saudy H.S., Wehbe N., Ben Hassine M., Al-Nemi R., Jaremko M., Emwas A.

Acknowledgements

The authors thank and appreciate Diener Company's assistance in helping us design a plasma device for treating seeds for use on medium agricultural lands. We urge the company to develop plasma devices that can process large amounts of seeds and are appropriate for cultivation in greater areas.

Supplementary materials

Supplementary Figure 1. Solid-state ^{13}C NMR spectra of control (blue) and one-minute treated samples (red). The figure shows very similar features of both spectra with slightly higher intensity at aliphatic and aromatic regions.
Supplementary Figure 2. High resolution XPS C 1s spectra of Misr samples.
Supplementary Figure 3. High resolution XPS C 1s spectra of Gemmeza-11 samples.

References

Abo, B.O.; Gao, M.; Wang, Y.; Wu, C.; Ma, H.; Wang, Q. 2019. Lignocellulosic biomass for bioethanol: An overview on pretreatment, hydrolysis and fermentation processes. Rev. Environ. Health 34, 57–68.

Alkordi, M.H., Haikal, R.R., Hassan, Y.S., Emwas, A.H., Belmabkhout, Y., 2015. Poly-functional porous-organic polymers to access functionality – co2 sorption energetic relationships.J. Mater. Chem. a, 3, 22584-22590.

Alves-Junior, C., Da Silva, D.L.S., Vitoriano, J.O., Barbalho, A.P.C.B. , De Sousa, R.C., 2020. The water path in plasma-treated leucaena seeds. Seed Sci. Res. 30, 13-20.

Attia, S. K., Elgendy, A.T. , Rizk, S.A., 2019. Efficient green synthesis of antioxidant azacoumarin dye bearing spiro-pyrrolidine for enhancing electro-optical properties of perovskite solar cells. J. Mole. Struc. 1184, 583-592.

Baraka, A., Sheashea, M., Gado, K., Rizk, S., Abuzalat, O., 2023. Designing of zn (ii)-based novel coordination polymer (cp): synthesis, characterization, and heavy metal removal from water. App. Organomet. Chem. 37, e6940-e6940.

Boerjan, W., Ralph, J., Baucher, M., 2003. Lignin biosynthesis. *annual review of plant biology*, 54, 519-546.

Case, S.D.C., McNamara, N.P., Reay, D.S., Whitaker, J., 2012. The effect of biochar addition on N_2O and CO_2 emissions from a sandy loam soil – the role of soil aeration. Soil Biology and Biochemistry, 51: 125–134. https://doi.org/10.1016/j.soilbio.2012.03.017

Chandra, K., Al-Harthi, S., Almulhim, F., Emwas, A. H., Jaremko, Ł., Jaremko, M. 2021a. The robust nmr toolbox for metabolomics. Mole. Omics 17, 719-724.

Chandra, K., Al-Harthi, S., Sukumaran, S., Almulhim, F., Emwas, A. H., Atreya, H. S., Jaremko, Ł. , Jaremko, M., 2021b. Nmr-based metabolomics with enhanced sensitivity. RSC Adv. 11, 8694-8700.

Chandra, M.R.G.S., Madakka, M., 2019. Chapter 11—Comparative Biochemistry and Kinetics of Microbial Lignocellulolytic Enzymes. In Recent Developments in Applied Microbiology and Biochemistry; Buddolla, V., Ed.; Academic Press: Cambridge, MA, USA. 147–159.

Chisca, S., Duong, P.H.H., Emwas, A.H., Sougrat, R., Nunes, S.P., 2014. Crosslinked copolyazoles with a zwitterionic structure for organic solvent resistant membranes. Pol. Chem 6, 543-554.

Dauwe, R., Roulard, R., Ramos, M., Thiombiano, B., Mesnard, F., Gontier, E., Jamali, A., 2021. Etching of the seed cuticle by cold plasma shortens imbibitional leakage in linum usitatissimum l. Indust. Crops Prod. 167, 113536-113536.

Davin, L.B., Lewis, N.G., 2005. lignin primary structures and dirigent sites. 16, 407-415.

El-Bially, M.E., Saudy, H.S., Hashem, F.A., El-Gabry, Y.A., Shahin, M.G., 2022a. Salicylic acid as a tolerance inducer of drought stress on sunflower grown in sandy soil. Gesun. Pflanz. 74, 603-613.

El-Bially, M.E., Saudy, H.S., El-Metwally, I.M., Shahin, M.G., 2022b. Sunflower response to application of l-ascorbate under thermal stress associated with different sowing dates. Gesun. Pflanz. 74, 87-96.

El-Hashash, M. A. , Rizk, S. A. 2017. One-pot synthesis of novel spirooxindoles as antibacterial and antioxidant agents. *journal of heterocyclic chemistry*, 54, 1776-1784.

El-Hashash, M. A., Rizk, S. A., El-Badawy, A. A. 2018. Ultrasonic aptitude of regioselective reaction of 6-bromo-spiro-3,1-benzoxazinone2,1′-isobenzofuran-3′,4-dione towards some electrophilic and nucleophilic reagents. *journal of heterocyclic chemistry*, 55, 2090-2098.

El-Mageed, T. A. A., Mekdad, A. A. A., Rady, M. O. A., Abdelbaky, A. S., Saudy, H. S. , Shaaban, A. 2022. Physio-biochemical and agronomic changes of two sugar beet cultivars grown in saline soil as influenced by potassium fertilizer. *journal of soil science and plant nutrition*, 22, 3636-3654.

El-Metwally, I. M. , Saudy, H. S. 2021a. Interactional impacts of drought and weed stresses on nutritional status of seeds and water use efficiency of peanut plants grown in arid conditions. *gesunde pflanzen,* 73**,** 407-416.

El-Metwally, I. M. , Saudy, H. S. 2021b. Interactive application of zinc and herbicides affects broad-leaved weeds, nutrient uptake, and yield in rice. *journal of soil science and plant nutrition,* 21**,** 238-248.

El–Metwally, I., Geries, L. , Saudy, H. 2021. Interactive effect of soil mulching and irrigation regime on yield, irrigation water use efficiency and weeds of trickle–irrigated onion. *archives of agronomy and soil science,* 68**,** 1103-1116.

Elgendy, A. T. 2019. Plasma boundary of nonlinear sheath dynamics for arbitrary waveforms in capacitive discharge. *journal of physics: conference series,* 1253**,** 012010-012010.

Elgendy, A. T. 2021. A Global Model Of The Collisional Plasma Boundary Sheath Using Step Model. *ieee transactions on plasma science,* 49**,** 711-717.

Elgendy, A. T., Alyousef, H. A. , Ahmed, K. M. 2022. New achievement of the global sheath-bulk model for the collisionless radio-frequency using in scale industries. *heliyon,* 8**,** e12264-e12264.

Elgendy, A. T., Basfer, N. M. , Al-Harbi, N. 2023. A collisional global sheath – bulk model of argon plasma for semiconductor scale manufacturing. *alexandria engineering journal,* 67**,** 437-446.

Emwas, A. H., Szczepski, K., Poulson, B. G., Chandra, K., Mckay, R. T., Dhahri, M., Alahmari, F., Jaremko, L., Lachowicz, J. I. , Jaremko, M. 2020. Nmr as a “gold standard” method in drug design and discovery. *molecules,* 25.

Felber, R., Leifeld, J., Horák, J., Neftel, A., 2014. N_2O emission reduction with green waste biochar: comparison of laboratory and field experiment. European Journal of Soil Science, 65: 128–138. https://doi.org/10.1111/ejss.12093

Gao, W. , Fatehi, P. 2019. Lignin for polymer and nanoparticle production: current status and challenges. *the canadian journal of chemical engineering,* 97**,** 2827-2842.

Ge, Y. , Li, Z. 2018. Application of lignin and its derivatives in adsorption of heavy metal ions in water: a review. *acs sustainable chemistry and engineering,* 6**,** 7181-7192.

Geng, W., Narron, R., Jiang, X., Pawlak, J.J., Chang, H.M., Park, S., Jameel, H., Venditti, R.A., 2019. The influence of lignin content and structure on hemicellulose alkaline extraction for non-wood and hardwood lignocellulosic biomass. Cellulose. 26, 3219–3230.

Han, L., Patil, S., Boehm, D., Milosavljević, V., Cullen, P. J. , Bourke, P. 2016. Mechanisms of inactivation by high-voltage atmospheric cold plasma differ for escherichia coli and staphylococcus aureus. *applied and environmental microbiology,* 82**,** 450-458.

Holc, M., Gselman, P., Primc, G., Vesel, A., Mozetič, M. , Recek, N. 2022. Wettability and water uptake improvement in plasma-treated alfalfa seeds. *agriculture 2022, vol. 12, page 96,* 12**,** 96-96.

Holc, M., Mozetič, M., Recek, N., Primc, G., Vesel, A., Zaplotnik, R. , Gselman, P. 2021. Wettability increase in plasma-treated agricultural seeds and its relation to germination improvement. *agronomy 2021, vol. 11, page 1467,* 11**,** 1467-1467.

Iles, J., 2015. Circular Economy—The Forgotten Low-Carbon Vector. Available online: https://circulatenews.org (accessed on 2 December 2015)

Jeevan Kumar, S. P., Rajendra Prasad, S., Banerjee, R. , Thammineni, C. 2015. Seed birth to death: dual functions of reactive oxygen species in seed physiology. *annals of botany,* 116, 663-668.

Kammann, C., Ratering, S., Eckhard, C., Müller, C., 2012. Biochar and hydrochar effects on greenhouse gas (carbon dioxide, nitrous oxide, and methane) fluxes from soils. Journal of Environmental Quality, 41 (4): 1052–1066.https://doi.org/10.2134/jeq2011.0132

Khalid Buraa, M., Obaid, A., Hussan Ali, H., Lan, Y., Al, Di, L., Zhang, J., Zhang, X., Elgendy, A. T. , Abdallah, T. 2019. Cancer therapy system based on gold nanoparticle / cold plasma via stimulated singlet oxygen production. *journal of physics: conference series,* 1253, 012003-012003.

Khan, M. T., Busch, M., Molina, V. G., Emwas, A.-H., Aubry, C. , Croue, J.-P. 2014. How different is the composition of the fouling layer of wastewater reuse and seawater desalination ro membranes? *water research,* 59, 271-282.

Kwan, E. E. , Huang, S. G. 2008. Structural elucidation with nmr spectroscopy: Practical strategies for organic chemists. *european journal of organic chemistry,* 2008, 2671-2688.

Li, S., Winters, H., Jeong, S., Emwas, A.-H., Vigneswaran, S. , Amy, G. L. 2016. Marine bacterial transparent exopolymer particles (tep) and tep precursors: characterization and ro fouling potential. *desalination,* 379, 68-74.

Liu, Z. H., Hao, N., Shinde, S., Pu, Y., Kang, X., Ragauskas, A. J. , Yuan, J. S. 2019. Defining lignin nanoparticle properties through tailored lignin reactivity by sequential organosolv fragmentation approach (sofa). *green chemistry,* 21, 245-260.

Ma, C. Y., Wang, H. M., Wen, J. L., Shi, Q., Wang, S. F., Yuan, T. Q. , Sun, R. C. 2020. Structural elucidation of lignin macromolecule from abaca during alkaline hydrogen peroxide delignification. *international journal of biological macromolecules,* 144, 596-602.

Mildaziene, V., Ivankov, A., Pauzaite, G., Naucienė, Z., Zukiene, R., Degutyte-Fomins, L., Pukalskas, A., Venskutonis, P. R., Filatova, I. , Lyushkevich, V. 2021. Seed treatment with cold plasma and electromagnetic field induces changes in red clover root growth dynamics, flavonoid exudation, and activates nodulation. *plasma processes and polymers,* 18, 2000160-2000160.

Mildaziene, V., Sera, B., 2022. Effects of non-thermal plasma treatment on plant physiological and biochemical processes. *plants 2022, vol. 11, page 1018,* 11, 1018-1018.

Mubarak, M., Salem, E.M.M., Kenawey, M.K.M., Saudy, H.S., 2021. Changes in calcareous soil activity, nutrient availability, and corn productivity due to the integrated effect of straw mulch and irrigation regimes. *journal of soil science and plant nutrition,* 21, 2020-2031.

Nassar, A.E., El-Aswar, E.I., Rizk, S.A., Gaber, S.E.S., Jahin, H.S., 2023. Microwave-assisted hydrothermal preparation of magnetic hydrochar for the removal of organophosphorus insecticides from aqueous solutions. *separation and purification technology,* 306, 122569-122569.

Pérez-Pizá, M.C., Prevosto, I., Grijalba, P.E., Zilli, C.G., Cejas, E., Mancinelli, B , Balestrasse, K.B., 2019. Improvement of growth and yield of soybean plants through the application of non-thermal plasmas to seeds with different health status. *heliyon,* 5, e01495-e01495.

Rizk, S.A., Abdelwahab, S S. , El-Badawy, A.A., 2019. Design, regiospecific green synthesis, chemical computational analysis, and antimicrobial evaluation of novel phthalazine heterocycles. *journal of heterocyclic chemistry,* 56, 2347-2357.

Rizk, S.A., Elsayed, G.A. , El-Hashash, M.A., 2018. One-pot synthesis, spectroscopic characterization and dft study of novel 8-azacoumarin derivatives as eco-friendly insecticidal agents. *journal of the iranian chemical society,* 15**,** 2093-2105.

Salem, E.M.M., Kenawey, K.M.M., Saudy, H.S., Mubarak, M., 2021. Soil mulching and deficit irrigation effect on sustainability of nutrients availability and uptake, and productivity of maize grown in calcareous soils. *communications in soil science and plant analysis,* 52**,** 1745-1761.

Saudy, H., Noureldin, N., Mubarak, M., Fares, W., Elsayed, M., 2019. cultivar selection as a tool for managing soil phosphorus and faba bean yield sustainability. *archives of agronomy and soil science,* 66**,** 414-425.

Saudy, H.S., El–Metwally, I.M., Shahin, M.G., 2021a. Co–application effect of herbicides and micronutrients on weeds and nutrient uptake in flooded irrigated rice: does it have a synergistic or an antagonistic effect? *crop protection,* 149**,** 105755-105755.

Saudy, H.S., Abd El-Samad, G.A., El-Temsah, M.E., El-Gabry,Y.A., 2021b. Effect of iron, zinc, and manganese nano-form mixture on the micronutrient recovery efficiency and seed yield response index of sesame genotypes. *journal of soil science and plant nutrition 2021 22:1,* 22**,** 732-742.

Scholtz, V., Jirešová, J., Šerá, B., Julák, J. 2021., A review of microbial decontamination of cereals by non-thermal plasma. *foods 2021, vol. 10, page 2927,* 10**,** 2927-2927.

Šerá, B., Vanková, R., Roháček, K., Šerý, M., Hayashi, N., Wang, D., Koga, K., 2021. Gliding arc plasma treatment of maize (zea mays l.) grains promotes seed germination and early growth, affecting hormone pools, but not significantly photosynthetic parameters. *agronomy 2021, vol. 11, page 2066,* 11**,** 2066-2066.

Shapira, Y., Bormashenko, E., Drori, E., 2019. Pre-germination plasma treatment of seeds does not alter cotyledon dna structure, nor phenotype and phenology of tomato and pepper plants. *biochemical and biophysical research communications,* 519**,** 512-517.

Söylemez, M. A., Özer, K., Ozer, D., 2021. Biofibers and their composites for industrial applications. *in:* inamuddin, t. a. (ed.). john wiley , sons, ltd.

Starič, P., Vogel-Mikuš, K., Mozetič, M., Junkar, I., 2020. Effects of nonthermal plasma on morphology, genetics and physiology of seeds: a review. *plants 2020, vol. 9, page 1736,* 9**,** 1736-1736.

Ten Bosch, L., Pfohl, K., Avramidis, G., Wieneke, S., Viöl, W., Karlovsky, P., 2017. Plasma-based degradation of mycotoxins produced by fusarium, aspergillus and alternaria species. *toxins 2017, vol. 9, page 97,* 9**,** 97-97.

Zahoranová, A., Henselová, M., Hudecová, D., Kaliňáková, B., Kováčik, D., Medvecká, V., Černák, M., 2016. Effect of Cold Atmospheric Pressure Plasma on the Wheat Seedlings Vigor and on the Inactivation of Microorganisms on the Seeds Surface. *Plasma Chemistry and Plasma Processing,* 36**,** 397-414.

Rizhiya, E.Y., Boitsova, L.V., Buchkina, N.P., Panova, G.G., 2011. The influence of crop residues with different C:N ratios on the N_2O emission from a loamy sand soddypodzolic soil. Eurasian Soil Science, 44: 1144. https://doi.org/10.1134/S1064229311100115

Toma, Y., Hatano, R., 2007. Effect of crop residue C:N ratio on N_2O emissions from Gray Lowland soil in Mikasa, Hokkaido, Japan. Soil Science and Plant Nutrition, 53 (2): 198–205. https://doi.org/10.1111/j.1747-0765.2007.00125.x

Table 1 Seedling growth rate of wheat cultivars (Misr-1 and Gemmeza-11) results at different plasma exposure times and its correlation coefficient.

Variable	Plasma treatment (time / min)	Seedling growth rate	Correlation Coefficient	Contact area on seed surface (A/ μm^2)
Misr-1 cultivar				
	No Treatment	Normal	0.2-0.4	101837
	1 min	Improved	0.6-0.75	113596
	2 min	Better improvement	0.75-1.00	68762
	3 min	Improved	0.6-0.75	89745
	4 min	Progress	0.4-0.6	137546
Gemmeza-11 cultivar				
	No Treatment	Normal	0.2-0.4	83456
	1 min	Improved	0.6-0.75	76892
	2 min	Better improvement	0.75-1.00	112564
	3 min	Improved	0.4-0.6	84535
	4 min	Progress	0.4-0.6	141002

Table 2. Physiological traits of wheat cultivars (Misr-1 and Gemmeza-11) produced under different plasma exposures in a greenhouse.

Samples	Ch-a (mg/g)	Ch-b (mg/g)	Total chl.	Carotenoid(mg/g)	MDA (nmol/gFW)	H_2O_2 (mM g–1 FW)
Misr-1 cultivar						
Misr -1 D(00)	0.71±0.06	0.54±0.08	1.25±0.02	0.28±0.01	23.37±3.36	0.319±0.04
Misr -1 D(22)	0.76±0.01	0.33±0.01	1.09±0.01	0.29±0.02	24.35±3.31	0.385±0.07
Misr -1 D(33)	0.65±0.11	0.42±0.08	1.07±0.03	0.23±0.06	31.32±0.85	0.712±0.02
Gemmeza-11 cultivar						
Gemmeza-11 D(00)	0.80±0.03	0.29±0.01	1.09±0.03	0.32±0.02	26.95±1.67	0.510±0.04
Gemmeza-11 D(22)	0.91±0.01	0.50±0.01	1.41±0.01	0.26±0.01	17.97±2.38	0.429±0.03
Gemmeza-11 D(33)	0.93±0.01	0.60±0.02	1.53±0.02	0.25±0.03	15.80±1.87	0.249±0.01

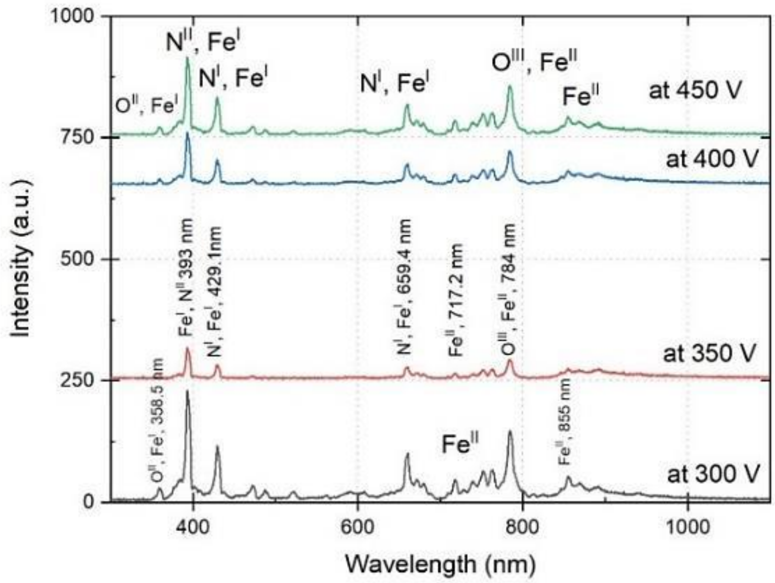

Figure 1. *Spectroscopic analysis of AC plasma of air during coating at different applied voltages. (Left) The panel shows the effect of the applied voltage on plasma intensity and composition. As expected, the intensity and diversity of species generated increased with increased voltage, which is also seen in the panel on the right.*

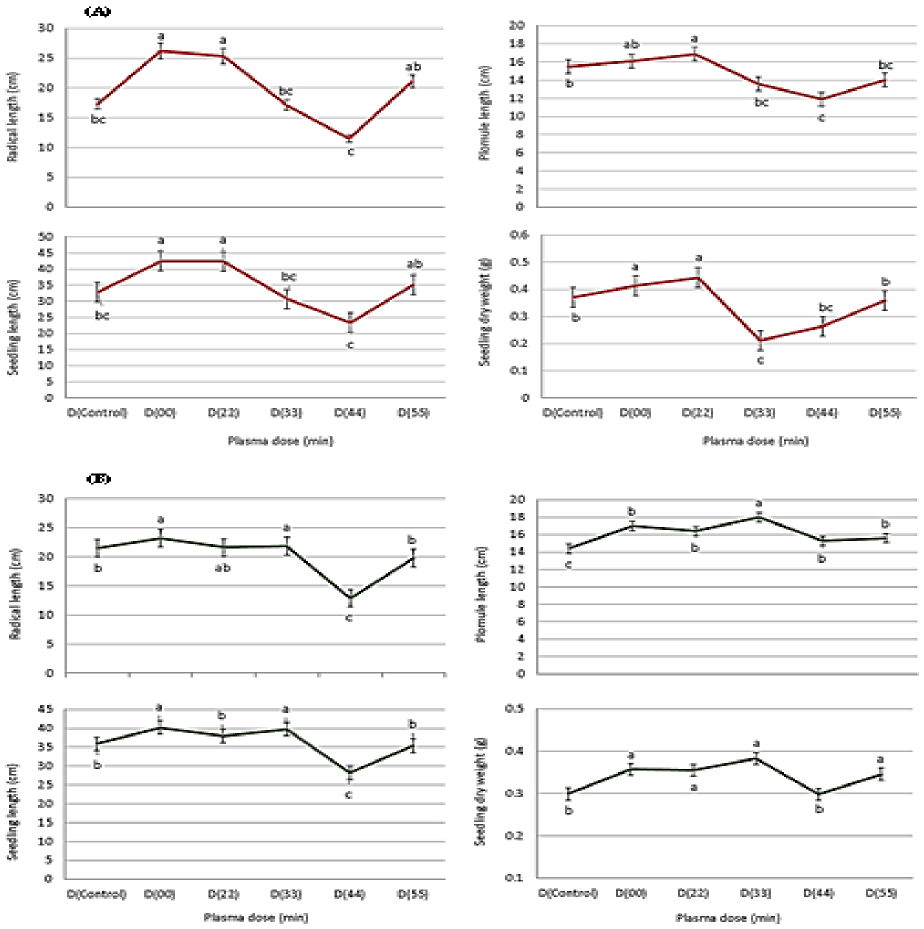

Figure 2. *Seedling growth of Misr-1 (A) and Gemmeza-11 (B) wheat cultivars as affected by plasma dose treatments measured after one month of growth in a petri dish Values are the mean of 3 replicates ± standard errors. Points with different letters are statistically significant at p ≤0.05*

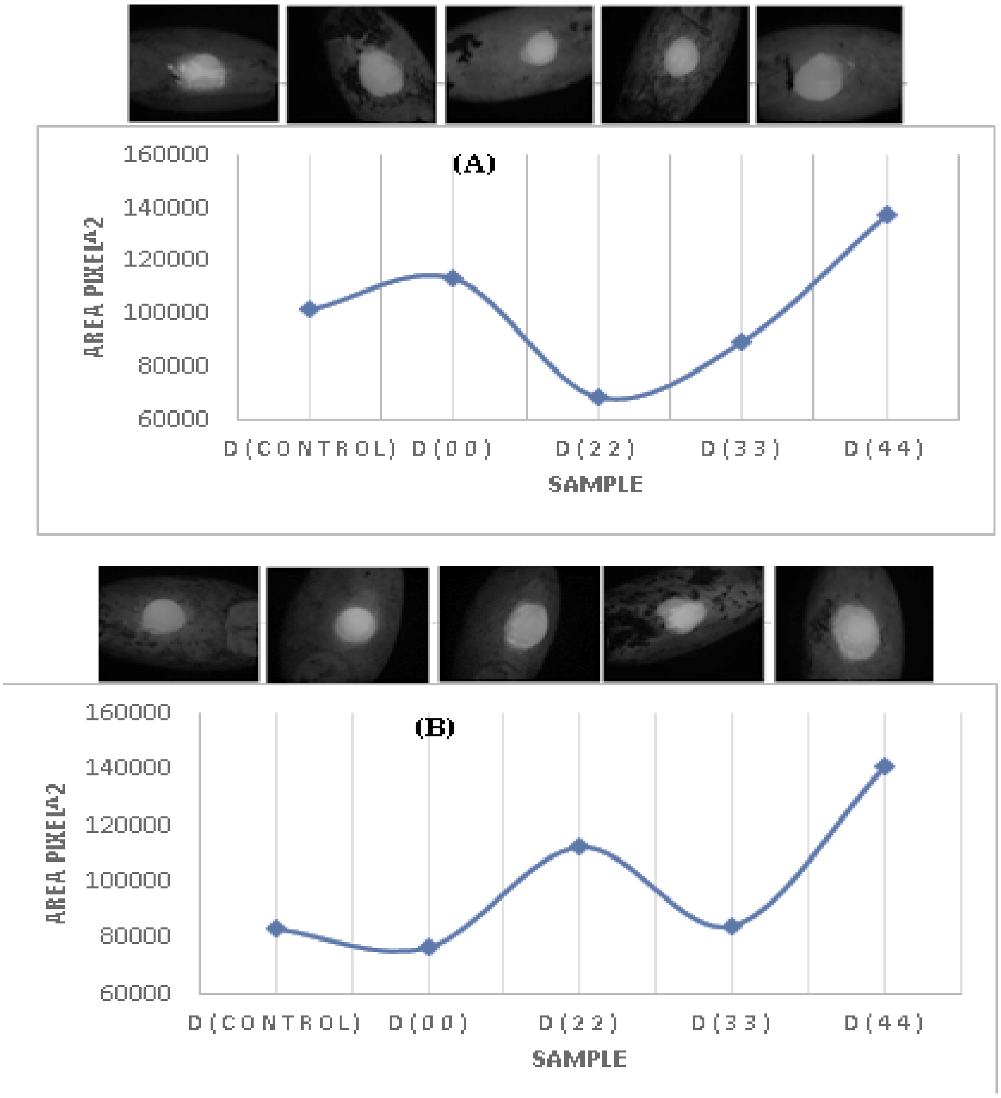

Figure 3. *Relationship between the contact area of water droplets on the surface of Misr-1 (A) and Gemmeza-11 (B) wheat seed (area) and different NTP doses (1 min: D(control), 2 min: D(00), 3 min: D(22), 4 min: D(33), 5 min: D(44)).*

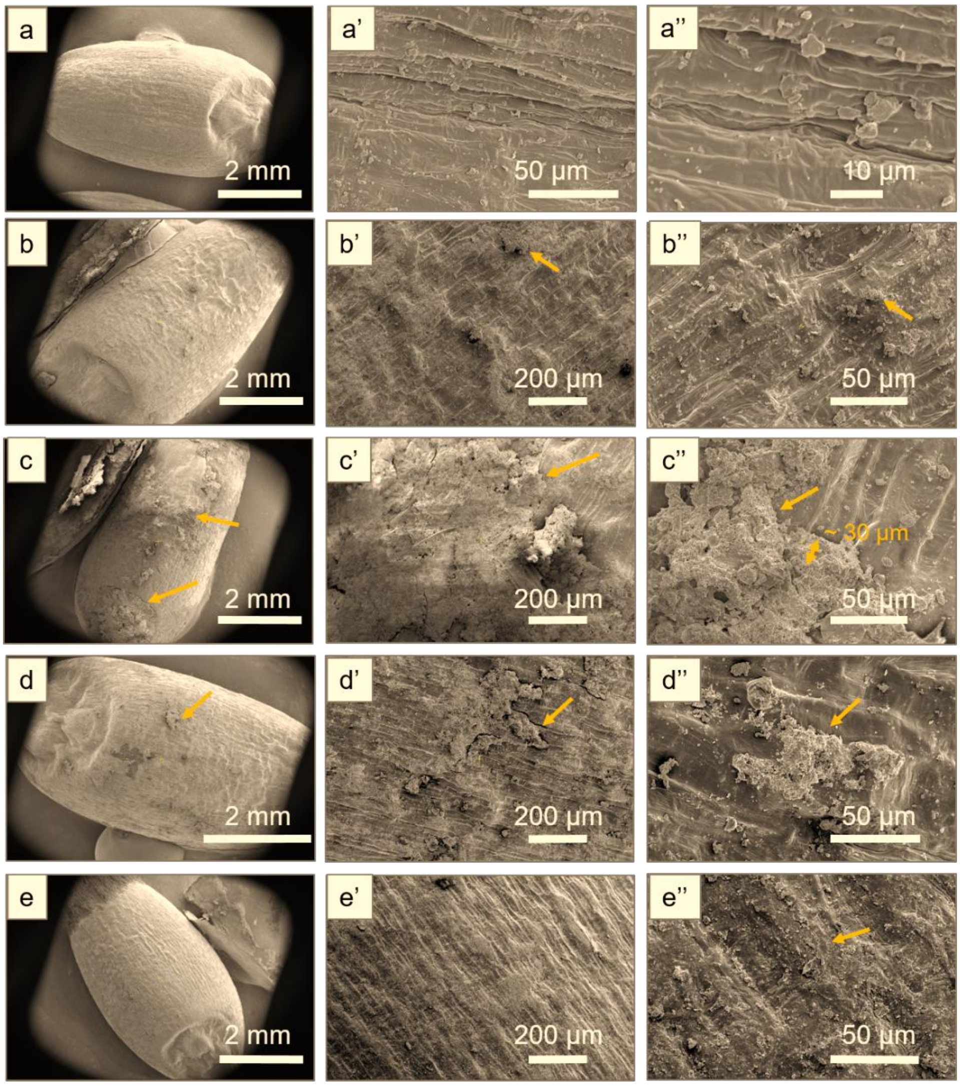

Figure 4: *SEM images of Misr samples:* ***(a-a'')*** *D control,* ***(b-b'')*** *D(00),* ***(c-c'')*** *D(22),* ***(d-d'')*** *D(33), and* ***(e-e'')*** *D(44). The images are shown at different scale as indicated.*

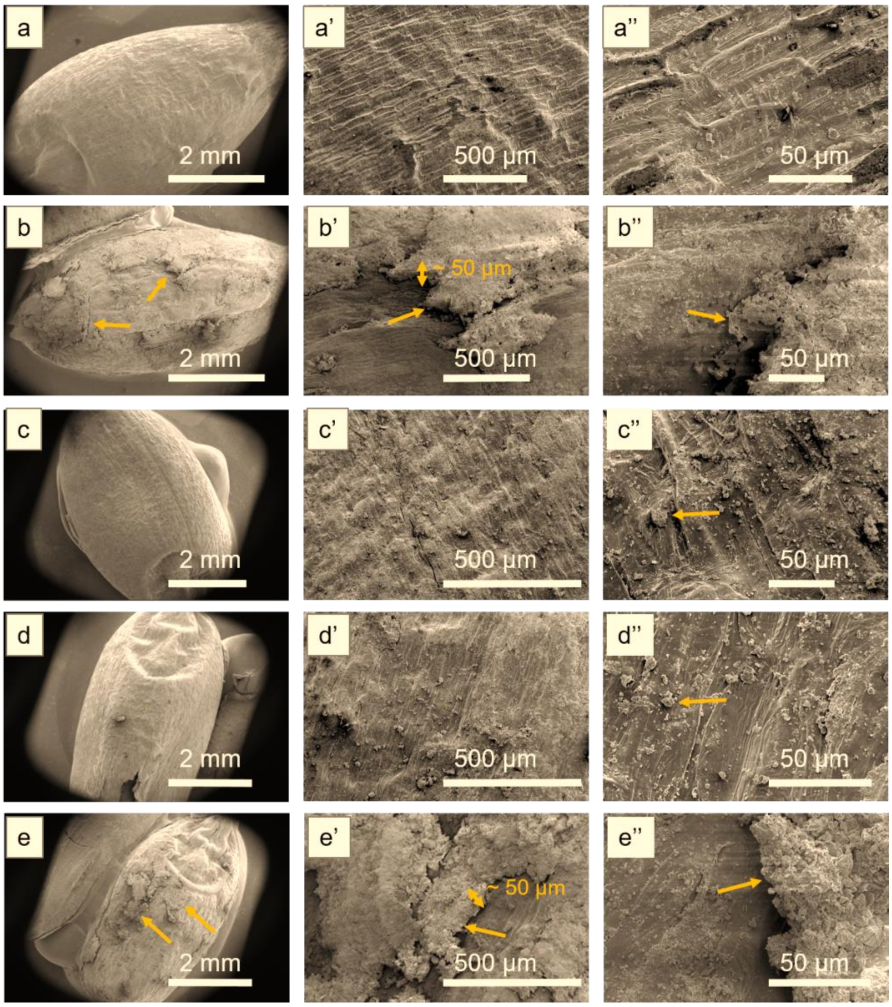

Figure 5: *SEM images of Gemmeza-11 samples:* ***(a-a'')*** *D control,* ***(b-b'')*** *D(00),* ***(c-c'')*** *D(22),* ***(d-d'')*** *D(33), and* ***(e-e'')*** *D(44). The images are shown at different scale as indicated.*

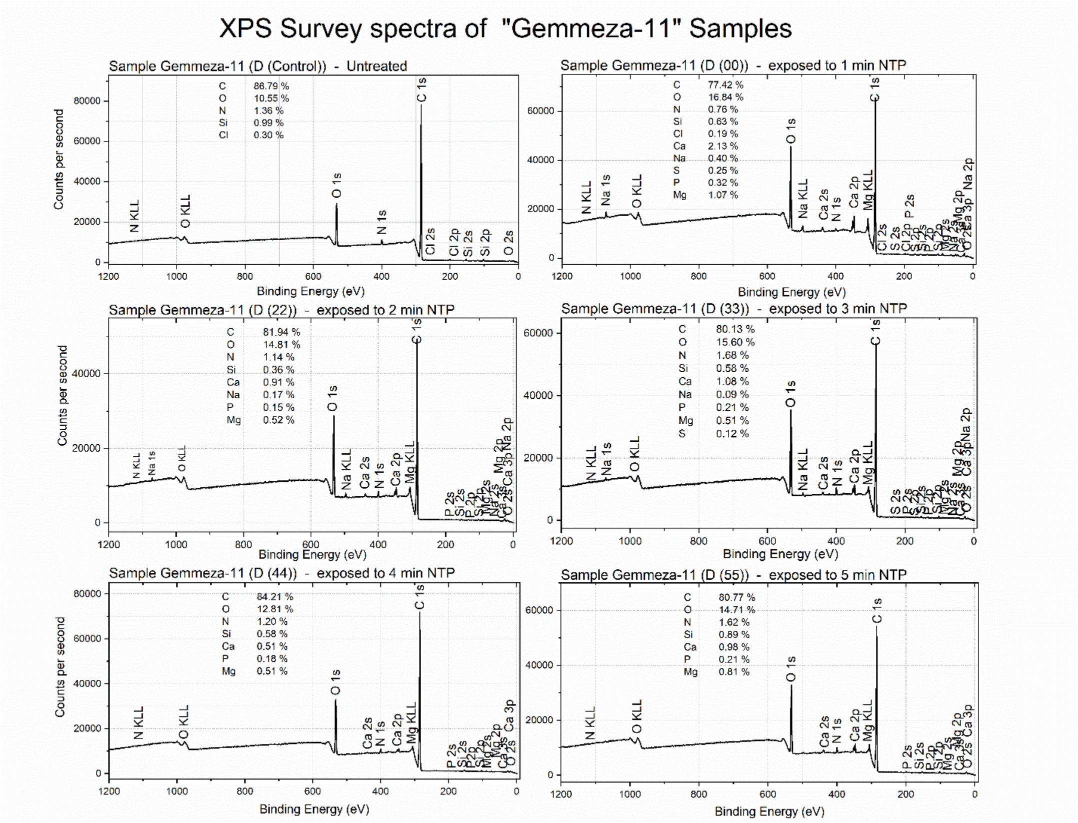

Figure 6. *XPS spectra of Gemmeza-11 seed samples treated under different conditions (D(00), D(11), D(22), D(33) and D(44), as well as the control sample.*

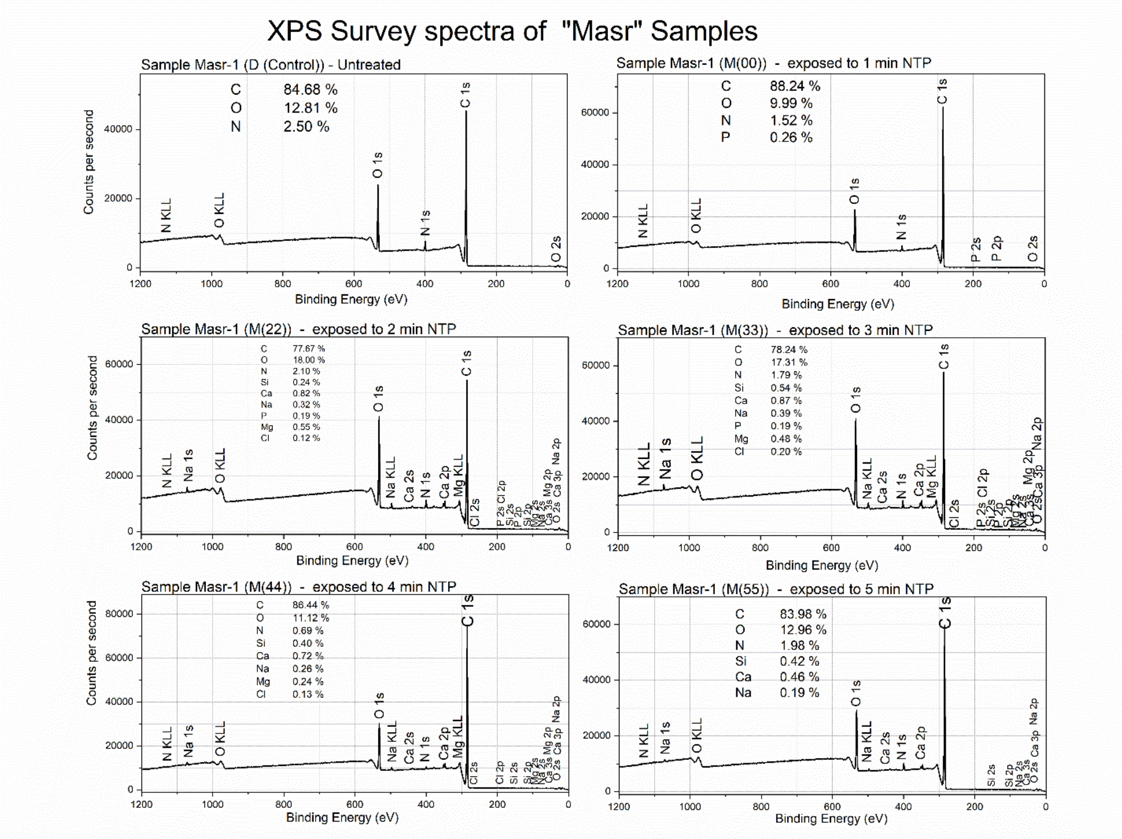

Figure 7. *The XPS survey spectra of treated and control Misr-1 Not Masr samples. D(00), D(11), D(22), D(33) and D(44) not M(00), M(11), M(22), M(33) and M(44), please add D(44) analysis.*

Click here to access/download
Supporting Information
Plasma Supporting Materials.docx
