# Supplementary material for: Undergoing lignin-coated seeds to cold plasma to enhance the growth of wheat seedlings and obtain future outcome under stressed ecosystems
Source: PLoS One. 2024 Sep 24;19(9):e0308269. doi: 10.1371/journal.pone.0308269 (PMC11421780; doi:10.1371/journal.pone.0308269)
Supplement: Attachment — Submitted filename: Reviewer 1.docx [file pone.0308269.s005.docx]

**Undergoing Lignin-Coated Seeds to Cold Plasma to Enhance the Growth of Wheat Seedlings and Obtain Future Outcome under Stressed Ecosystems**

**Reviewer 1**

1. The final paragraph of the introduction in this study seems to emphasize methodology rather than objectives. Instead of explaining what and how something was done, this section should identify a gap and outline the objectives aimed at addressing that gap.
2. In the "Plant Material" subsection of the Materials and Methods section, only the characteristics of the plants and the locations where they were obtained should be discussed, but instead, methods were mentioned.
3. The Materials and Methods section should be divided into two subsections. If the Material section is 2.1, then the Methods section will be 2.2, and the subsequent sections will be numbered sequentially as 2.2.1, 2.2.2, 2.2.3, and so on (e.g., Chemical Lignocellulosic Biomass Materials, Plasma Treatment, Wettability Measurements, etc.).
4. Also this text should be in the statistical analysis section "Based on the randomized complete block design in four replicates, plasma treatment and wheat cultivar were considered as fixed effects, and replications (blocks) were considered as random effects
5. There is no need to repeat the methods in the Results section. The Results section should only include the findings and reference the tables and figures where these findings are presented.
6. Do not give method in results section such as “After one month, radical length, plumule length, seedling length, and seedling dry weight were measured”.
7. Line 269 has an empty square, which is unclear.
8. Given that solid-state NMR has not previously been used for wheat seed analysis, we had to develop a sample preparation procedure that would retain seed integrity and allow for accurate acquisition of the NMR data (see sample preparation description in Materials and Methods section). No citation? References?
9. Please put a comma before each "and."
10. The Results section has turned into a Materials and Methods section, with numerous method descriptions.
11. The discussion section is weak. There is no attempt to establish a connection between the literature and the findings. A few pieces of literature are mentioned first, followed by the findings, but similarities and differences are not discussed. The discussion section, which is the most important part and can contribute the most to science, prompting researchers to think, innovate, and generate new ideas, has been kept very brief in this publication. Although this was a study done with effort, it requires major revision. There is no real discussion in the discussion section. I do not believe that co-authors have read this paper. At least one of them should know that this paper, in its current form, should not be submitted to the PLOS ONE journal. It is likely that a very junior academic wrote this paper and added the names of other colleagues. It is not recommended for publication in its current form. I request that it be sent back to me after major revision.
