# Supplementary material for: Undergoing lignin-coated seeds to cold plasma to enhance the growth of wheat seedlings and obtain future outcome under stressed ecosystems
Source: PLoS One. 2024 Sep 24;19(9):e0308269. doi: 10.1371/journal.pone.0308269 (PMC11421780; doi:10.1371/journal.pone.0308269)
Supplement: Attachment — Submitted filename: Response to Reviewers.docx [file pone.0308269.s006.docx]

**Author Response**

**Dear Respected Editor and Reviewers,**

Thank you so much for reviewing and editing our manuscript. We thoroughly revised and modified the manuscript guided with the useful editorial and reviewer’s comments and suggestions. Please find the revised and modified manuscript, in addition to the response letter replying to point-by-point received comments from editor and reviewers.

| **Comments** | **Author Response** | **Notes** |
| --- | --- | --- |
| **Reviewer #1, all required correction Highlighted in yellow** | |  |
| The study presents a novel and innovative method in addressing the challenges of seed germination in stressed ecosystems by introducing cold plasma as a treatment to improve seed vigor. The physiological analyses presented in the paper provide a comprehensive understanding of the effects of cold plasma treatment and lignin coating on seed germination and growth. The study evaluated various parameters, including seed germination percentage, growth rate, and antioxidant activity to assess seed vigor following the cold plasma and lignin coating treatments. These analyses provide significant insights into the mechanisms involved in the enhancement of wheat seed growth in stressed ecosystems. | Thank you very much for the motivational opinion |  |
| Regarding the seed germination experiment, the manuscript does not specify the seed germination protocol utilized. Therefore, it is unclear if ISTA or AOSA guidelines were followed, which might lead to concerns about replicability and comparability of the results. The manuscript should clarify the applied protocol and provide specific details for replicability purposes. | Please note that the current study involved two levels of experimentations (Lab and Greenhouse))  Concerning the first level of experimentation (Lab test):  As you know ISTA guidelines are applied on the laboratory tests for seeds. Exactly, we used the rules prescribed by ISTA to estimate the germination test. We mentioned more details | See lines: 186-188 |
| The sample size used for the vigor assessment is limited to five seeds per treatment. While this is an acceptable number for preliminary screening of germination and growth potential, it is considered small for solid scientific conclusions. The manuscript should justify the reasoning behind the use of such a small sample size and provide further analyses, including standard deviation and statistical significance to support the presented data. | The second level experimentation:  **For Greenhouse trial, the use of 5 seeds to be planted in small pots** (30-cm diameter) has been determined based on previous studies. This trial aimed mainly to obtain plant materials for assessing some physiological parameters which require small leaves samples (the leaves of three plants are so sufficient).  The obtained results statistically analyzed as shown in Table 2 | See Table 2 |
| The manuscript does not explicitly identify prominent environmental stressors considered during the study, which limits the applicability and generalizability of the obtained results. This makes it challenging to infer the relevance of the study's findings to actual ecological systems. The manuscript should specify the significant environmental stressors considered during the study and contextualize the results with their relevance to the relevant ecosystem. | In green house experiment the seed have been planted in salt-affected soil. As we described the procedures of greenhouse experiment, the plasma-treated seeds were planted in plastic pots of 30-cm diameter which filled with 6 kg saline soil (salinity level was 5.5 dS m^-1^). It is well known that the salinity level in normal soils do not exceed 3.0 dS m^-1^. Accordingly, the experiment included stressed soil environment. | See lines: 201-202 |
| In conclusion, the "Cold Plasma Treatment of Lignin-Coated Wheat Seeds Enhances Growth in Stressed Ecosystems" study presents a novel and innovative approach to improve seed germination and growth in stressed ecosystems. The study's physiological analyses provide useful insights into the effects of cold plasma treatment and lignin coating on seed vigor. While the study has some limitations, including concerns about the sample size and lack of clarity regarding the used seed germination protocol and environmental stressors, the research presents a crucial contribution to the field of seed germination under challenging ecological conditions. | Thank you so much for valuable comments. We believe we have addressed and answered all the questions asked by Reviewer #1  We would appreciate your other suggestions in the next review round.  . |  |
| **Reviewer #2, all required correction Highlighted in blue** | |  |
| 1. The final paragraph of the introduction in this study seems to emphasize methodology rather than objectives. Instead of explaining what and how something was done, this section should identify a gap and outline the objectives aimed at addressing that gap | This paragraph rephrased | See lines: 81-86 |
| 2. In the "Plant Material" subsection of the Materials and Methods section, only the characteristics of the plants and the locations where they were obtained should be discussed, but instead, methods were mentioned. | Clarified well | See Lines: 90-132 |
| 3. The Materials and Methods section should be divided into two subsections. If the Material section is 2.1, then the Methods section will be 2.2, and the subsequent sections will be numbered sequentially as 2.2.1, 2.2.2, 2.2.3, and so on (e.g., Chemical Lignocellulosic Biomass Materials, Plasma Treatment, Wettability Measurements, etc.). | Materials and Methods section modified as suggested | See Materials and Methods section |
| 4. Also this text should be in the statistical analysis section "Based on the randomized complete block design in four replicates, plasma treatment and wheat cultivar were considered as fixed effects, and replications (blocks) were considered as random effects | Ok, this text already mentioned | See lines: 208-210 |
| 5. There is no need to repeat the methods in the Results section. The Results section should only include the findings and reference the tables and figures where these findings are presented. | You have all right, Thanks.  **Under sub-title "Effect of plasma dose on wheat seedling growth** " the repeated wordings omitted | See the Results section |
| 6. Do not give method in results section such as “After one month, radical length, plumule length, seedling length, and seedling dry weight were measured”. | OK, methods which mentioned in Results removed | See the Results section |
| 7. Line 269 has an empty square, which is unclear. | Corrected  | See line: 250 |
| 8. Given that solid-state NMR has not previously been used for wheat seed analysis, we had to develop a sample preparation procedure that would retain seed integrity and allow for accurate acquisition of the NMR data (see sample preparation description in Materials and Methods section). No citation? References? | The reference of assessing NMR added | See lines:180, 182 |
| 9. Please put a comma before each "and." | Done |  |
| 10. The Results section has turned into a Materials and Methods section, with numerous method descriptions. | Results section completely rewritten | See the Results section |
| 11. The discussion section is weak. There is no attempt to establish a connection between the literature and the findings. A few pieces of literature are mentioned first, followed by the findings, but similarities and differences are not discussed. The discussion section, which is the most important part and can contribute the most to science, prompting researchers to think, innovate, and generate new ideas, has been kept very brief in this publication. Although this was a study done with effort, it requires major revision. There is no real discussion in the discussion section. I do not believe that co-authors have read this paper. At least one of them should know that this paper, in its current form, should not be submitted to the PLOS ONE journal. It is likely that a very junior academic wrote this paper and added the names of other colleagues. It is not recommended for publication in its current form. I request that it be sent back to me after major revision. | Discussion section improved. Kindly, note that the current study is one of the preliminary attempts to link among several multidisciplinary (physics, agriculture, organic chemistry and biochemistry). Therefore, the available related studies area rare. Also, since this study had different specializations, one author cannot individually prepare the manuscript. Each co-author performed a specific act as shown in author contributions statement. | See Discussion section |
| **Editor comments all required correction Highlighted in green** | |  |
| 1. Please ensure that your manuscript meets PLOS ONE's style requirements, including those for file naming. | Based on the PLOS ONE's style requirements, we re-prepared our manuscript | See the manuscript |
| 2. Thank you for stating the following financial disclosure: "The research was funded via Science & Technology Development Fund (STDF), Ministry of Scientific Research Egypt, for financial support and providing required research facilities through the approved project, ID: IG-43568."  Please state what role the funders took in the study.  If the funders had no role, please state: ""The funders had no role in study design, data collection and analysis, decision to publish, or preparation of the manuscript."" | The funders had no role in study design, data collection and analysis, decision to publish, or preparation of the manuscript.  This statement add in "Funding Information’ | See lines: 421-422 |
| 3. We note that the grant information you provided in the ‘Funding Information’ and ‘Financial Disclosure’ sections do not match.  When you resubmit, please ensure that you provide the correct grant numbers for the awards you received for your study in the ‘Funding Information’ section. | OK, thanks |  |
| Please include this amended Role of Funder statement in your cover letter; we will change the online submission form on your behalf. | Done | See cover letter |
| There are significant concerns about the manuscript's grammar, usage, and overall readability. We, therefore, request that you revise the text to fix the grammatical errors and improve the overall readability of the text. | The text revised carefully and all grammatical errors removed. Also, the overall readability of the text improved. | See the manuscript text. |
